# Comparison of MRI and Histopathology with regard to Intramedullary Extent of Disease in Bone Sarcomas

**DOI:** 10.1155/2019/7385470

**Published:** 2019-11-29

**Authors:** Ashish Gulia, Ajay Puri, T. S. Subi, Srinath M. Gupta, S. L. Juvekar, Bharat Rekhi

**Affiliations:** ^1^Bone and Soft Tissue Services, Department of Surgical Oncology, Tata Memorial Hospital, HBNI, Mumbai 400012, India; ^2^Department of Surgical Oncology, Rajagiri Hospital, Aluva, Kerala 683112, India; ^3^Department of Radiology, National Cancer Institute, Nagpur 441108, India; ^4^Department of Surgical Pathology, Tata Memorial Hospital, HBNI, Mumbai 400012, India

## Abstract

In today's era, limb salvage surgery is the procedure of choice and current standard of care in appropriately selected patients of bone sarcomas. For adequate oncologic clearance, preoperative evaluation of the extent of tumor is mandatory. The present study was done to compare measurements of bone sarcomas (osteosarcoma, Ewing's sarcoma, and chondrosarcoma) as determined by magnetic resonance imaging (MRI) with the histopathological extent seen on resected specimens. We prospectively evaluated 100 consecutive patients with a diagnosis of bone sarcoma who underwent limb salvage surgery between May 2014 and December 2014. The maximum longitudinal (cranio-caudal) dimension of tumor on the noncontrast T1-WI sequence of MRI (irrespective of whether it was pre/postchemotherapy) was compared with the gross dimensions of the tumor on histopathology. The arithmetic mean difference, Wilcoxon signed-rank test, and Spearman's correlation analysis were used to test the differences and correlation between groups. Mean tumor size on MRI based on the largest extent on MRI was 12.1 ± 4.85 cm (mean ± standard deviation), while it was 10.77 ± 4.6 cm (mean ± standard deviation) on histopathology. In 79 cases, MRI overestimated the extent of disease; the mean was 1.79 cm with a standard deviation of 1.56 cm. When the disease extent was underestimated on MRI (13 cases), the mean was 0.58 cm with a standard deviation of 0.43 cm. In 8 cases (osteosarcoma (7), Ewing's sarcoma (1)), MRI measurement was equal to histopathology. The Spearman correlation analysis showed a high correlation of tumor length on histopathology with the MRI for all patients (*R* = 0.948, *P* < 0.0001). We thus conclude that MRI is accurate in delineating the extent of bone sarcomas. A margin of 2 cm from the maximum tumor extent is adequate to ensure appropriate surgical resection.

## 1. Introduction

Complete tumor removal is critical to achieve adequate disease control and provide optimum oncological outcomes in bone sarcomas. In today's era, limb salvage surgery has become the procedure of choice and the current standard of care in appropriately selected patients [1, 2]. For adequate oncologic clearance, preoperative evaluation of the extent of tumor is mandatory [3, 4]. Inadequate excision of tumor-bearing bone can result in tumor recurrence and contribute to poor oncological outcomes, and hence limb salvage should only be performed after detailed preoperative planning that ensures complete tumor removal [5]. Unnecessary resections can lead to compromised function and a higher incidence of reconstruction failure [6]. Presently, magnetic resonance imaging (MRI) is considered as the best imaging modality to detect the extent of tumor involvement [7, 8]. Accurate estimation of tumor extent on MRI is the key to plan optimal resection margins [9–13]. Currently, there are limited studies comparing MRI with postresection histopathological measurements.

The present study compares measurements in bone sarcomas (osteosarcoma, Ewing's sarcoma, and chondrosarcoma) as determined by the MRI with the histopathological extent seen on resected specimens.

## 2. Materials and Methods

We prospectively evaluated 100 consecutive patients with a diagnosis of bone sarcoma who underwent limb salvage surgery between May 2014 and December 2014. Institutional ethics committee approval was obtained for the study. The study included patients with a confirmed histopathological diagnosis of osteosarcoma/Ewing's sarcoma/chondrosarcoma who underwent limb salvage surgery at our centre. We excluded patients who underwent reimplantation of bone after extracorporeal radiation therapy where margins and tumor extent could not be assessed on post-resection histopathology (HP) [14].

At presentation, all patients underwent local disease evaluation with a plain radiograph in two perpendicular planes and MRI of the local site imaging the entire length of the involved bone. After confirmation of histopathological diagnosis and staging, the patients were treated as per standard hospital protocol (neoadjuvant chemotherapy for osteosarcoma and Ewing's sarcoma) and upfront surgery for chondrosarcoma. After completion of neoadjuvant therapy which lasted for approximately 3 months, a repeat MRI was done for all patients. The last MRI was performed within 6 weeks of index surgery. Both pre- and postchemotherapy MRI images were reviewed by a radiologist specialising in musculoskeletal oncology, and details of the tumor site, size, and maximum disease extent (intramedullary extent/periosteal reaction/soft tissue mass) were noted. For the purpose of this study, the maximum extent of disease as measured on noncontrast T1-weighted images was noted [15]. The maximum longitudinal (cranio-caudal) dimension of tumor on MRI (irrespective of whether it was a prechemotherapy or postchemotherapy MRI) was considered as this is the extent which determines the level for tumor resection [15]. The imaging was performed on 1.5T system (Signa, GE). T1-WI coronal sequences were performed using a body coil with a repetition time range/echo time range of 300–600 ms/4–6 ms and 4 mm section thickness and 1 mm spacing.

**Figure 1:**
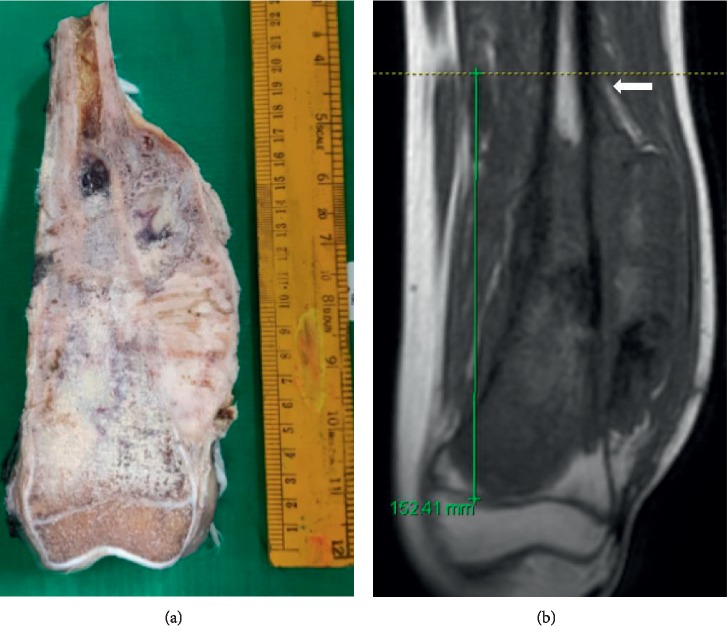
Tumor length measurements in the case of distal femur osteosarcoma on the gross specimen (a) 16.5 cm and noncontrast T1-weighted coronal MRI (b) 15.2 cm. Note that the white arrow indicates the periosteal reaction and soft tissue component exceed the intramedullary tumor extent.

**Figure 2:**
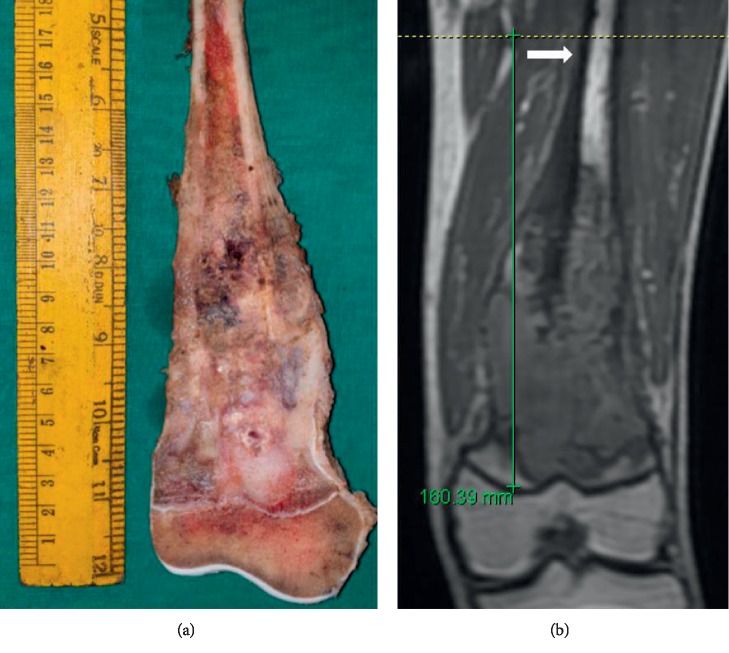
Tumor length measurements in the case of distal femur osteosarcoma on the gross specimen (a) 14 cm and noncontrast T1-weighted coronal MRI (b) 16 cm. Note that the white arrow indicates the periosteal reaction exceeds the intramedullary tumor extent.

After surgical resection, the excised specimens were grossed by a pathologist specialising in musculoskeletal oncology. After bisecting the specimen longitudinally, the gross dimensions of the tumor were recorded. The extent of disease involvement as seen on the specimen was measured using a millimetre scale and was confirmed by histopathology sections that evaluated maximum disease extent. The radiological and histopathological dimensions were correlated. All the radiological and histopathological assessments were done by the same radiologist and pathologist who specialised in musculoskeletal oncology and were aware of the ongoing study. Examples of measurements are shown in Figures 1 and 2.

The arithmetic mean difference, Wilcoxon signed-rank test, and Spearman's correlation analysis were used to test the differences and correlation between groups.

## 3. Results

**Figure 3:**
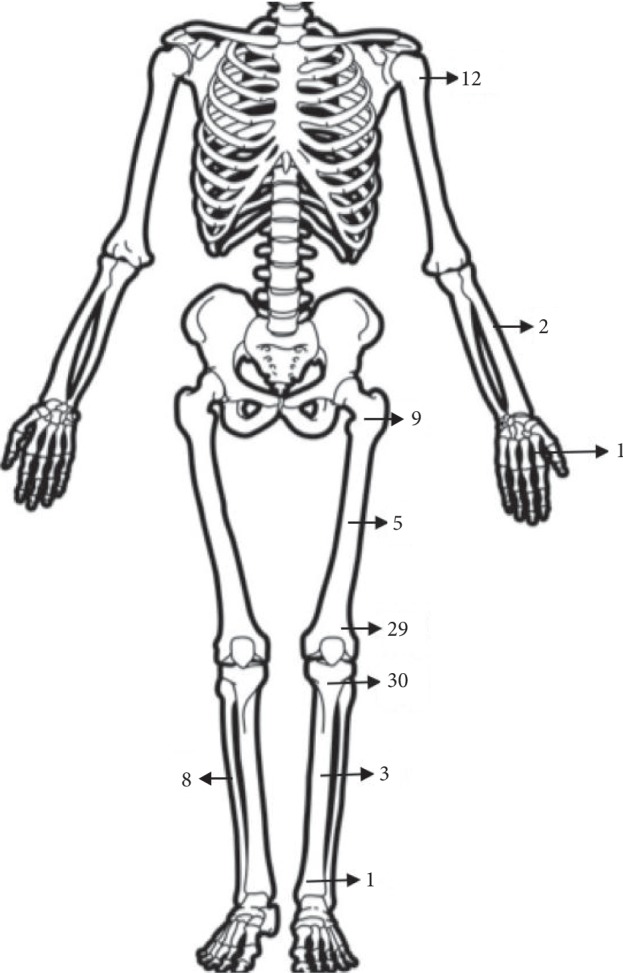
Distribution as per site.

A total of 100 cases were included in the study; 73 osteosarcomas, 20 Ewing's sarcomas, and 7 chondrosarcomas. Proximal tibia (30) and distal femur (29) were the most common sites involved (Figure 3). 89 patients received chemotherapy.

Mean tumor size on MRI based on the largest extent on either pre/postchemotherapy MRI was 12.1 ± 4.85 cm (mean ± standard deviation), while it was 10.77 ± 4.6 cm (mean ± standard deviation) on histopathology. The mean difference and standard deviation of subgroups are given in Table 1. In 8 cases (osteosarcoma (7), Ewing's sarcoma (1)), MRI measurement was equal to histopathology.

The Spearman correlation analysis showed a high correlation of tumor length on histopathology with the MRI for all patients (*R* = 0.948, *P* < 0.0001).

## 4. Discussion

With improved surgical techniques and effective neoadjuvant therapy, the current rate of limb salvage in bone sarcomas is 85% to 90% [16, 17]. An adequate surgical resection provides the best chance for local disease control, which contributes to better disease-related survival in bone sarcomas [10, 11, 18]. Balancing the desire to retain best possible function necessitates that the surgical excision must have adequate oncologic clearance while avoiding unnecessary excessive resection. Prior to the era of current advanced imaging modalities, intraoperative evaluation of the bone marrow by frozen section was the norm in surgical treatment of bone sarcomas [19]. This is time consuming, needs the availability of a dedicated pathologist for assessing the sample during surgery, and adds an additional financial cost to treatment [20]. Advances in imaging have improved our ability to accurately assess the extent of tumor on MRI. This can help reduce or obviate intraoperative frozen section sampling [20]. Anderson et al. in a study of 142 patients concluded that frozen section can be omitted to determine the disease status at the osteotomy site [20] though they did suggest examination of the split gross specimen as an adjunct to clinical and radiological findings to ensure negative margins.

While there are a few published studies comparing the accuracy of imaging in determining the extent of disease in osteosarcoma, studies comparing the same for Ewing's sarcoma and chondrosarcoma are scarce [8, 19, 21–28]. Gillepsy et al. [22] compared CT and MRI in 17 cases of osteosarcoma and determined that MRI is extremely accurate in assessing the intraosseous extent with a difference of 4.9 ± 4.3 mm. In a subgroup of five specimens with an identical plane of section, the average difference reduced to 1.8 mm ± 1.6. O'Flanagan's study [26] comparing CT, MRI, and bone scans to estimate the extent of tumor in resected specimens determined that an MRI gave the most accurate results. Onikul et al. documented a mean difference in MRI and postoperative gross specimen measurements within 2 cm [8].

In Han et al.'s series, restricted to only osteosarcoma cases, the maximum underestimation was 0.9 cm and the maximum over estimation was 3.4 cm [23]. Tao's study [25] excluded tumors involving thin bones like the radius, ulna and fibula, whereas we have included all tumors in 100 consecutive cases. In their study, estimation of tumor length was done on gross specimens whereas we had histopathology confirmation confirming the pathologic extent of disease as well. Tao et al. showed a median difference of 2 mm (range: 0.1 cm to 1.5 cm) where the radiological disease extent was overestimated and a median difference of 5 mm (range: 0.1 cm to 1.8 cm) when the radiological disease extent was underestimated [25]. In our study when MRI overestimated the extent of disease, the mean was 1.79 cm with a standard deviation of 1.56 cm. When the disease extent was underestimated on MRI, the mean was 0.58 cm with a standard deviation of 0.43 cm.

While in Tao's series, the maximum underestimation was 1.8 cm, and it was 1.5 cm in our series. Thus, a margin of 2 cm from the maximum tumor extent can be considered an adequate safety margin to avoid intralesional resections. While a 2 cm margin is ideal, occasionally there will be instances when a surgeon may choose to have a lesser margin in order to preserve a growth plate or an articular joint. It may be advisable to augment radiologic estimations of disease extent with intraoperative frozen section sampling or examination of split gross specimens in these cases.

## 5. Conclusions

The findings of the present study reiterate the fact that MRI is accurate in delineating the extent of bone sarcomas. A margin of 2 cm from the maximum tumor extent is adequate and can avoid unnecessary lengthy resections. In the current era of imaging, frozen section sampling after resection in bone sarcomas may be omitted without compromising oncologic clearance in cases where a 2 cm margin is possible.

## Figures and Tables

**Table 1:** Subgroup analysis.

	Mean difference ± SD (cm)	Minimum (cm)	Maximum (cm)
MRI length *>* HP (79)	1.79 ± 1.56	0.10	4.00
Mean difference in tumor length in cases where dimension in MRI is more than HP			
Osteosarcoma (54)	1.8 ± 1.2	0.10	4.00
Ewing's sarcoma (19)	1.98 ± 1.10	0.30	4.00
Chondrosarcoma (6)	1.12 ± 0.79	0.50	2.50
HP size *>* MRI (13)	0.58 ± 0.43	0.10	1.50
Mean difference in tumor length in cases where dimension in HP is more than MRI			
Osteosarcoma (12)	0.58 ± 0.45	0.10	1.50
Chondrosacoma (1)	0.6 ± 0.6	0.60	0.60

## Data Availability

The datasets of the current study are available from the corresponding author upon request.

## References

[1] Meyer J. S., Mackenzie W. (2004). Malignant bone tumors and limb-salvage surgery in children. *Pediatric Radiology*.

[2] Wallack S. T., Wisner E. R., Werner J. A. (2002). Accuracy of magnetic resonance imaging for estimating intramedullary osteosarcoma extent in pre-operative planning of canine limb-salvage procedures. *Veterinary Radiology Ultrasound*.

[3] Reddy K. I. A., Wafa H., Gaston C. L. (2015). Does amputation offer any survival benefit over limb salvage in osteosarcoma patients with poor chemonecrosis and close margins?. *Bone & Joint Journal*.

[4] Anderson M. E. (2016). Update on survival in osteosarcoma. *Orthopedic Clinics of North America*.

[5] Bacci G., Ferrari S., Lari S. (2002). Osteosarcoma of the limb. Amputation or limb salvage in patients treated by neoadjuvant chemotherapy. *Journal of Bone and Joint Surgery*.

[6] Mavrogenis A. F., Coll-Mesa L., Gonzalez-Gaitan M. (2011). Criteria and outcome of limb salvage surgery. *Journal of the Balkan Union of Oncology*.

[7] Bloem J. L., Taminiau A. H., Eulderink F., Hermans J., Pauwels E. K. (1988). Radiologic staging of primary bone sarcoma: MR imaging, scintigraphy, angiography, and CT correlated with pathologic examination. *Radiology*.

[8] Onikul E., Fletcher B. D., Parham D. M., Chen G. (1996). Accuracy of MR imaging for estimating intraosseous extent of osteosarcoma. *American Journal of Roentgenology*.

[9] Kawaguchi N., Ahmed A. R., Matsumoto S., Manabe J., Matsushita Y. (2004). The concept of curative margin in surgery for bone and soft tissue sarcoma. *Clinical Orthopaedics and Related Research*.

[10] Bertrand T. E., Cruz A., Binitie O., Cheong D., Letson G. D. (2016). Do surgical margins affect local recurrence and survival in extremity, nonmetastatic, high-grade osteosarcoma?. *Clinical Orthopaedics and Related Research®*.

[11] Li X., Moretti V. M., Ashana A. O., Lackman R. D. (2012). Impact of close surgical margin on local recurrence and survival in osteosarcoma. *International Orthopaedics*.

[12] Bellanova L., Paul L., Docquier P.-L. (2013). Surgical guides (patient-specific instruments) for pediatric tibial bone sarcoma resection and allograft reconstruction. *Sarcoma*.

[13] Xu M., Xu S., Yu X. (2014). Marginal resection for osteosarcoma with effective neoadjuvant chemotherapy: long-term outcomes. *World Journal of Surgical Oncology*.

[14] Puri A., Byregowda S., Gulia A., Patil V., Crasto S., Laskar S. (2018). Reconstructing diaphyseal tumors using radiated (50 Gy) autogenous tumor bone graft. *Journal of Surgical*.

[15] Thévenin-Lemoine C., Destombes L., Vial J. (2018). Planning for bone excision in ewing sarcoma: post-chemotherapy MRI more accurate than pre-chemotherapy MRI assessment. *The Journal of Bone and Joint Surgery*.

[16] Halperin E., Constine L., Tarbell N., Kun L. (2012). Pediatric radiation oncology. https://books.google.com/books?hl=en&lr=&id=UMKN7qerq3wC&oi=fnd&pg=PA184&ots=SdlLVkDq9g&sig=yFonYACVRZPvqoT-CQMjZwQaMH0.

[17] Gulia A. (2016). Osteosarcoma—a clandestine enigma. *Journal of Bone & Soft Tissue Tumors*.

[18] Chou A. J., Merola P. R., Wexler L. H. (2005). Treatment of osteosarcoma at first recurrence after contemporary therapy. *Cancer*.

[19] Meyer M. S., Spanier S. S., Moser M., Scarborough M. T. (1999). Evaluating marrow margins for resection of osteosarcoma. A modern approach. *Clinical Orthopaedics and Related Research*.

[20] Anderson M. E., Miller P. E., van Nostrand K., Vargas S. O. (2014). Frozen section versus gross examination for bone marrow margin assessment during sarcoma resection. *Clinical Orthopaedics and Related Research®*.

[21] Bellanova L., Schubert T., Cartiaux O. (2014). MRI-based assessment of safe margins in tumor surgery. *Sarcoma*.

[22] Gillespy T., Manfrini M., Ruggieri P., Spanier S. S., Pettersson H., Springfield D. S. (1988). Staging of intraosseous extent of osteosarcoma: correlation of preoperative CT and MR imaging with pathologic macroslides. *Radiology*.

[23] Han G., Wang Y., Bi W.-Z. (2012). Magnetic resonance imaging is appropriate for determining the osteotomy plane for appendicular osteosarcoma after neoadjuvant chemotherapy. *Medical Oncology*.

[24] Hao Y., Zhang Y., Yang Z., Li X., Yang Q., Li J. (2008). The accuracy of magnetic resonance imaging in determining the osteotomy plane in osteosarcoma. *Orthopedics*.

[25] Jin T., Deng Z.-P., Liu W.-F., Xu H.-R., Li Y., Niu X.-H. (2017). Magnetic resonance imaging for the assessment of long bone tumors. *Chinese Medical Journal*.

[26] O’Flanagan S. J., Stack J. P., McGee H. M., Dervan P., Hurson B. (1991). Imaging of intramedullary tumour spread in osteosarcoma. A comparison of techniques. *Journal of Bone and Joint Surgery*.

[27] Putta T., Gibikote S., Madhuri V., Walter N. (2019). Accuracy of various MRI sequences in determining the tumour margin in musculoskeletal tumours. *Polish Journal of Radiology*.

[28] Thompson M. J., Shapton J. C., Punt S. E., Johnson C. N., Conrad E. U. (2018). MRI identification of the osseous extent of pediatric bone sarcomas. *Clinical Orthopaedics and Related Research*.

